# DeepBCTPred: deep learning-based prediction of bladder cancer tissues from endoscopic images

**DOI:** 10.1093/bioadv/vbag087

**Published:** 2026-03-26

**Authors:** Md Muhaiminul Islam Nafi, Khandokar Md Rahat Hossain

**Affiliations:** Department of Computer Science and Engineering, Bangladesh University of Engineering and Technology, Dhaka 1000, Bangladesh; Department of Computer Science and Engineering, United International University (UIU), Dhaka 1212, Bangladesh; Department of Computer Science and Engineering, Bangladesh University of Engineering and Technology, Dhaka 1000, Bangladesh; Department of Computer Science and Engineering, United International University (UIU), Dhaka 1212, Bangladesh

## Abstract

**Motivation:**

Bladder cancer is one of the most prevalent malignancies worldwide, affecting the tissues of the urinary bladder and posing a significant threat to patient survival and quality of life. Accurate classification of bladder cancer tissue is critical for early diagnosis and patient survival, yet conventional methods suffer from subjective interpretation and human error.

**Results:**

We propose DeepBCTPred, a novel deep learning framework that integrates handcrafted and learned features through a dual-branch architecture combining MobileNetV3 with a Feedforward Neural Network. Our approach incorporates Recursive Feature Elimination (RFE) for feature selection and employs a genetic algorithm-based image generation pipeline for optimal data selection. DeepBCTPred achieved superior performance with 98.74% recall, 99.45% specificity, and 97.96% F1-score on the test dataset, significantly outperforming existing state-of-the-art methods, achieving improvements ranging from 2% to 15% in recall, 1.3%–13.1% in F1-score, and 1.5%–16% in Matthews Correlation Coefficient (MCC). This framework demonstrates strong potential for clinical implementation in bladder cancer diagnosis and may be extensible to other cancer types for enhanced precision medicine applications.

**Availability and implementation:**

The training, validation, and test scripts are freely available at https://github.com/nafcoder/DeepBCTPred.

## 1 Introduction

Bladder cancer is one of the several types of cancer that affects the tissues of the urinary bladder. It ranks among the top 10 most prevalent cancers worldwide. It significantly affects the mortality rates and patient’s quality of life. There are two major forms of bladder cancer: Non-Muscle-Invasive Bladder Cancer (NMIBC) (Shelley *et al.* 2012) and Muscle-Invasive Bladder Cancer (MIBC) (Smith *et al.* 2013). NMIBC is seen in ∼70% to 80% of newly diagnosed cases. It usually affects the inner layers of the bladder wall (Álvarez-Maestro *et al.* 2021). This form is less aggressive but well known for its high recurrence rate, with up to 50% cases progressing to MIBC over time. On the other hand, MIBC spreads deeper into the bladder. It is associated with poorer forecasting where radical treatments like systemic therapies or cystectomy are often needed (Smith *et al.* 2013, Arcangeli *et al.* 2015).

Early detection with high accuracy of bladder cancer is essential to improve patient outcomes. However, traditional diagnostic methods have many challenges. Cystoscopy, the standard for diagnosis and monitoring, needs a direct visual examination of the bladder (Vining *et al.* 1996, Matulewicz *et al.* 2017, McCarthy 2021). Innovations like Narrow-Band Imaging (NBI) and Blue-Light Cystoscopy (BLC) have enhanced the diagnostic accuracy of conventional White-Light Imaging (WLI) (Ye *et al.* 2015, Sano *et al.* 2016). These methods are highly dependent on the expertise of the operator, thus becoming susceptible to the probability of unintentionally failing to detect some lesions. High-grade tumors are often challenging to distinguish from low-grade tumors (Karakök *et al.* 2001). These issues highlight the need for computational methods that can enhance diagnostic precision and consistency, thereby reducing the risk of recurrence and progression.

Early-stage bladder cancer tissues can be treated with comparatively less invasive surgery or therapy than advanced stages that require chemotherapy or immunotherapy (City of Hope 2024). Early and accurate diagnosis can prevent harmful effects. Advancements in imaging technologies like NBI offer enhanced contrast for tumor visualization. However, limitations still persist in detecting subtle lesion types for small and flat tumors (Sano *et al.* 2016). This has introduced the necessity of adopting artificial intelligence (AI)-based solutions, which provide automated, consistent, and highly accurate tissue classification. By integrating AI models during cystoscopic evaluations, diagnostic errors can be minimized. Thus, the outcome of the treatment will improve, which decreases the transition risks from NMIBC to MIBC (Ikeda *et al.* 2020, Negassi *et al.* 2020). To address these challenges, a publicly available dataset of endoscopic images, originally curated by Lazo *et al.* (2023), was leveraged by us. Images were obtained from NBI video data for three patients and WLI video data for the remaining patients. Following the classification framework established by the World Health Organization (WHO) and the International Society of Urological Pathology (ISUP), the dataset includes four categories: High-Grade Cancer (HGC), Low-Grade Cancer (LGC), Non-Suspicious Tissue (NST), and No Tumor Lesion (NTL), which encompasses inflammatory conditions such as cystitis. By utilizing this dataset, an AI-based approach aimed at improving bladder cancer tissue classification was developed by us. Our method integrates deep learning techniques with feature engineering to enhance diagnostic precision, ultimately reducing misclassification rates and supporting better clinical decision-making.

AI models such as deep learning (DL) models have revolutionized medical imaging, including various diagnostic tasks. Convolutional Neural Networks (CNNs) are recognized for their ability to extract complex features from visual data. They have become a cornerstone of diagnostic technologies (Chua 1997). Emerging architectures like Vision Transformers (ViTs) show significant promise in analyzing high-resolution images, which leverage attention mechanisms for superior feature representation (Han *et al.* 2023). Garapati *et al.* (2017) developed a computerized system to assess the bladder cancer stage using a dataset of 84 lesions from 76 Computed Tomography Urography (CTU) cases. The classifiers, such as Linear Discriminant Analysis (LDA), Neural Network (NN), Support Vector Machine (SVM) (Cortes 1995), and Random Forest (RF) (Breiman 2001), were evaluated using texture and morphological features. Ikeda *et al.* (2020) designed a GoogLeNet (Szegedy *et al.* 2015)-based AI model to improve the quality of bladder cancer diagnosis. Their dataset was 2102 cystoscopic images, consisting of 1671 images of normal tissue and 431 images of tumor lesions. Hashemi *et al.* (2021) proposed a method for evaluating cystoscopy images using a dataset of 720 images collected from a medical center. The method involved evaluating several models, including VGG-16 (Simonyan and Zisserman 2014), VGG-19 (Simonyan and Zisserman 2014), AlexNet (Krizhevsky *et al.* 2012), and ResNet (He *et al.* 2016). Among these, VGG-16 achieved the best performance. Yang *et al.* (2021) evaluated the performance of three different CNNs to recognize bladder tumors. The CNN models that were tested in their work were LeNet (LeCun *et al.* 1998), AlexNet, and GoogLeNet. Their dataset contained 1200 cystoscopic images with cancer and 1150 images without cancer. Ali *et al.* (2021) combined blue light cystoscopy with pre-trained neural networks [InceptionV3 (Antoni *et al.* 2017), MobileNetV2 (Sandler *et al.* 2018), ResNet50 (He *et al.* 2016) and VGG16] which identified flat and small lesions. Their dataset contained 216 blue light (BL) images acquired during Photodynamic Diagnosis (PDD)-guided Transurethral Resection of Bladder Tumor (TUR-BT). Lazo *et al.* (2023) introduced a semi-supervised Generative Adversarial Network (GAN)-based method for tissue classification in multi-domain datasets. Tiwari *et al.* (2023) presented a hybrid CNN model that integrates a limited number of handcrafted features, obtained through textural analysis and Bag-of-Visual Words (BoVW), with learned features. Handcrafted features refer to the properties or features extracted from the information present in the image itself, not from any machine learning (ML) or deep learning (DL) model. Devi *et al.* (2024) proposed a 17-layered deep CNN (17-DCNN) for endoscopic bladder cancer tissue classification. This model was benchmarked against DenseNet121 (Huang *et al.* 2017), ResNet152 (He *et al.* 2016), VGG19Net, and InceptionV3Net. They evaluated their models’ suitability for early diagnosis applications. Sunnetci *et al.* (2025) proposed multiple models for bladder cancer tissue classification. They introduced three separate models: a CNN-based deep learning model, a hybrid model that involved classifying learned features obtained from a CNN-based network with ML, and a Vision Transformer (ViT) model. Lutviana *et al.* (2025) built a CNN-based model for the same prediction task. Sathya *et al.* (2025) designed a model that used transfer learning with the ResNet50 model, which does feature extraction and classification at once. The dataset used in this study was from the work of (Lazo *et al.* 2023, Tiwari *et al.* 2023, Devi *et al.* 2024, Sunnetci *et al.* 2025, Lutviana *et al.* 2025, Sathya *et al.* 2025) also used the same dataset. The comparative analysis of bladder cancer classification methods is provided in Table 1.

**Table 1:** Comparative analysis of bladder cancer classification methods.

Study	Explored models and methodologies	Dataset
Garapati *et al.* (2017)	LDA, NN, SVM, RF	84 Lesions from 76 CTU cases with texture and morphological features
Ikeda *et al.* (2020)	GoogLeNet	2102 Cystoscopic images (1671 normal, 431 tumor lesions)
Hashemi *et al.* (2021)	VGG-16, VGG-19, AlexNet, ResNet	720 Cystoscopic images
Yang *et al.* (2021)	LeNet, AlexNet, GoogLeNet	1200 Cystoscopic images with cancer, 1150 images without cancer
Ali *et al.* (2021)	InceptionV3, MobileNetV2, ResNet50, VGG16	216 Blue Light images during TUR-BT (PDD-guided)
Lazo *et al.* (2023)	Semi-supervised GAN-based Model	Same as our study
Tiwari *et al.* (2023)	Hybrid CNN + Handcrafted Features	Same as our study
Devi *et al.* (2024)	Seventeen Layered Deep CNN, DenseNet121, ResNet152, VGG19Net, InceptionV3Net	Same as our study
Sunnetci *et al.* (2025)	CNN, Hybrid CNN+ML, ViT	Same as our study
Lutviana *et al.* (2025)	CNN	Same as our study
Sathya *et al.* (2025)	ResNet50	Same as our study

Although existing state-of-the-art (SOTA) methods have made significant advancements in bladder cancer tissue detection, there are still several gaps that limit their broad-scale adoption. One major limitation is the insufficient exploration of the integration of handcrafted features with deep learning models. This integration can potentially improve the performance of classification. The usage of traditional machine learning models for feature selection and data augmentation is still not explored. So, there is a potential chance that the already gained outcomes from the existing SOTA methods are suboptimal. Because feature selection techniques have been used in multiple research domains (Ullah *et al.* 2024a,b) to improve prediction performance. Additionally, the lack of comprehensive benchmarking of the state-of-the-art methods hampers a thorough evaluation of new approaches. It prevents a clear understanding of the relative strengths and weaknesses of the models. In this study, we performed feature integration and applied genetic algorithms, which have been used in various research domains (Akbar *et al.* 2025a,b).

In our study, a novel model, DeepBCTPred, was designed to predict bladder cancer tissues. Different handcrafted features and some prominent deep-learning architectures were utilized for prediction. Hybrid approaches like combining handcrafted features with deep learning-derived (CNN model-derived) features add an extra layer of robustness. Firstly, different handcrafted features were extracted, and Recursive Feature Elimination (RFE) was used for feature selection. To select the model that will be used for running RFE, 12 different traditional ML models were explored. Then, a novel genetic algorithm was employed with an image generation pipeline to augment new images in the existing original dataset. Various models that are based on CNNs were analyzed, and the best model was chosen from the validation results. Our model was rigorously tested on an independent test set, and it outperformed the existing state-of-the-art (SOTA) methods. To improve model interpretability and support real-world applications, Gradient-weighted Class Activation Mapping (Grad-CAM) was utilized to generate heatmaps.

The heatmaps and predictions generated from our proposed model can help doctors by highlighting specific regions of medical images that were most important in the model’s decision-making process. This allows the doctors to cross-check the highlighted regions. It provides an additional layer of assurance and reduces the chance of misdiagnosis. By integrating these aids, our proposed model can facilitate more accurate and precise decision-making in clinical practice.

The key contributions of DeepBCTPred in bladder cancer tissue prediction are the following:

- DeepBCTPred integrates handcrafted features with learned features in a hybrid architecture, combining MobileNetV3 (Howard *et al.* 2019) and a Feedforward Neural Network (FNN) to improve bladder cancer tissue classification.
- A new image generation pipeline was designed to augment the dataset, incorporating a series of transformations to create diversified images that help improve model generalization.
- A genetic algorithm was introduced to intelligently select the most informative augmented images, optimizing the dataset for better model performance and generalization.
- Handcrafted features extracted from the images were optimized using RFE, contributing to improved prediction accuracy by reducing redundant or irrelevant features.
- DeepBCTPred outperforms state-of-the-art (SOTA) methods in bladder cancer tissue prediction, achieving superior results in recall, specificity, and F1-score.
- To support real-world applications and improve interpretability, Grad-CAM was utilized to generate heatmaps that visualize the model’s decision-making process, aiding clinicians in understanding the predictions.

The sections of this paper are structured as follows: Section 2 summarizes the dataset used, the extraction of handcrafted features, feature selection techniques, performance metrics, image augmentation, and the model architecture with its configurations. Section 3 presents the experimental outcomes, including various plots for result analysis. Section 4 offers an overall summary, discusses limitations, and suggests future research directions. Lastly, Section 5 covers disclosures, acknowledgments, and data availability.

## 2 Materials and methods

**Figure 1:**
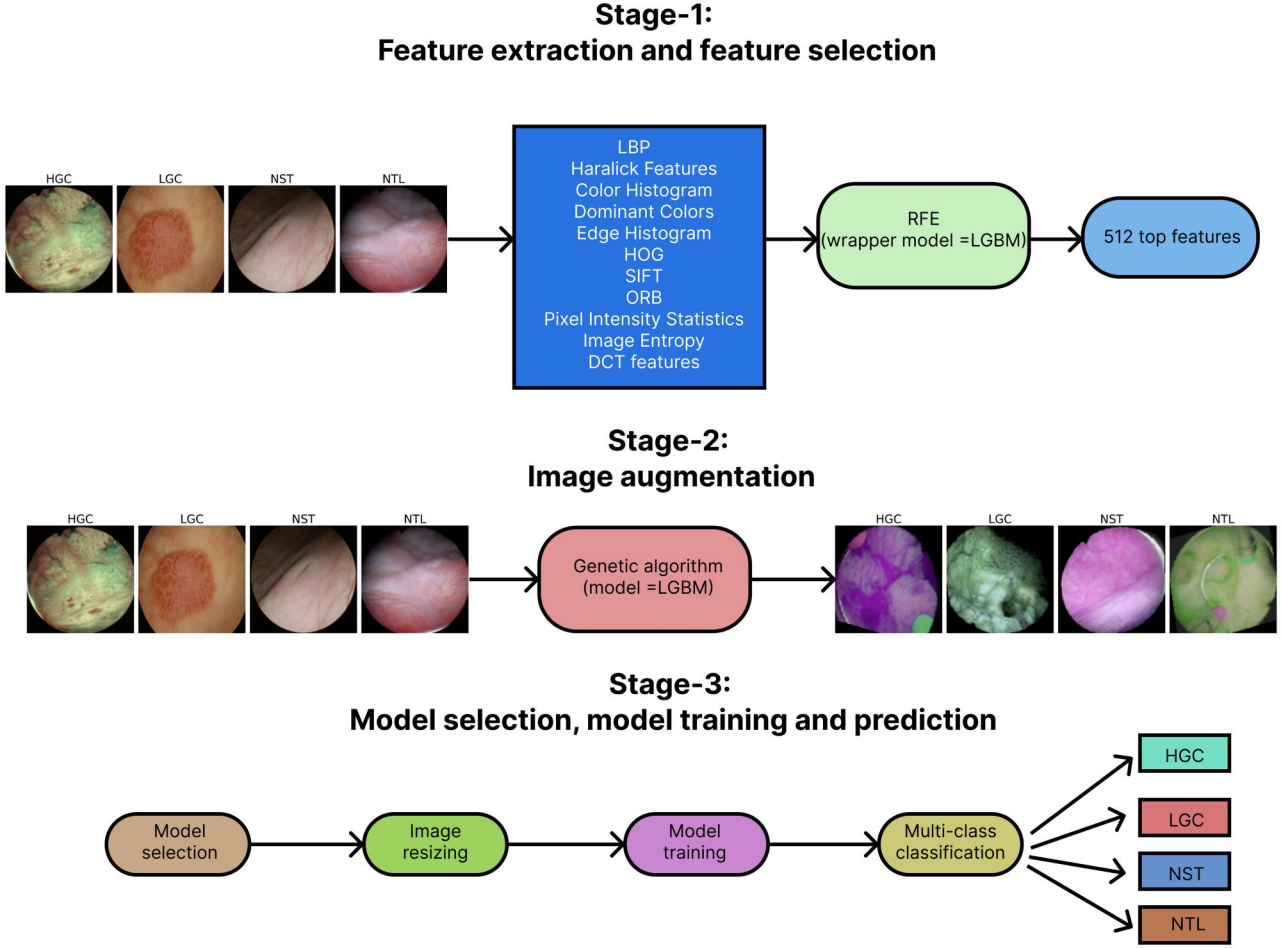
Overall workflow of the proposed DeepBCTPred framework for bladder cancer tissue prediction. The pipeline consists of three main stages: (1) Feature extraction and selection, where various handcrafted features (e.g. LBP, Haralick features, color histograms, SIFT, HOG, ORB, entropy, and DCT features) are extracted and the top 512 features are selected using Recursive Feature Elimination (RFE) with LGBM as the wrapper model; (2) Image augmentation, where a novel genetic algorithm–based strategy is employed to generate diverse augmented images, improving the dataset’s variability; and (3) Model selection, training, and prediction, where multiple architectures are benchmarked, the best-performing model is trained on the augmented dataset, and multi-class classification is performed across different bladder tissue types (HGC, LGC, NST, NTL).

In this section, the following descriptions are provided: dataset, handcrafted feature extraction, feature selection methods like RFE, image augmentation, framework, and configuration of the proposed model. The overall workflow is depicted in Fig. 1.

### 2.1 Dataset

The original dataset, retrieved from the works of Lazo *et al.* (2023), had endoscopic images captured during Transurethral Resection of Bladder Tumors (TURBT) (a procedure in which bladder tumors are surgically removed). As shown in Table 2, the dataset had 1754 images that were collected from 23 patients. These images were extracted from Narrow-Band Imaging (NBI) video data for three patients and White-Light Imaging (WLI) video data for the remaining patients. In line with the general classification of bladder cancer defined by the World Health Organization (WHO) and the International Society of Urological Pathology (ISUP), class labels High-Grade Cancer (HGC) and Low-Grade Cancer (LGC) were considered. Additionally, No Tumor Lesion (NTL), which includes cystitis induced by infections or other inflammatory agents, and Non-Suspicious Tissue (NST) were included in the class labels. All of the images were in Portable Network Graphics (PNG) format. This dataset contained 469, 647, 504, and 134 images for the classes HGC, LGC, NST, and NTL, respectively.

**Table 2:** Summary of the original dataset.

Dataset	Total images	HGC images	LGC images	NST images	NTL images
Original dataset	1754	469	647	504	134

The original dataset was randomly split in a 9:1 ratio, with the 10% portion designated as the **Test set**. The remaining 90% was further randomly divided in an 8:2 ratio, resulting in 80% being assigned as the **Training set** and 20% as the **Validation set**. New images were generated using the image generation pipeline, from which 500 images were selected through the genetic algorithm and referred to as the **D1 set**. The **Training** and **D1** sets were concatenated to form the **D2 set**. Similarly, the **Training** and **Validation** sets were combined and referred to as the **D3 set**. Additionally, the **Training**, **Validation**, and **D1** sets were merged and named the **D4 set**. A summary of the datasets is provided in Table 3.

**Table 3:** Summary of the datasets used in our study.

Dataset	Total images	HGC images	LGC images	NST images	NTL images
Training	1262	338	465	362	97
Validation	316	84	117	91	24
Test	176	47	65	51	13
D1 (augmentation)	500	128	208	138	26
D2 (training + D1)	1762	466	673	500	123
D3 (training + validation)	1578	422	582	453	121
D4 (training + validation + D1)	2078	550	790	591	147

### 2.2 Feature extraction and feature selection

Handcrafted features are derived by applying specific mathematical techniques or rules to extract useful patterns or properties from an image. The extraction techniques analyze pixel values and their relationships to extract meaningful information from the image. In our experiment, before extracting the features, the images were resized to dimensions of 128 × 128 pixels and normalized. A resolution of 224 × 224 was avoided to reduce the feature space dimension and maintain computational efficiency.

Feature groups refer to the collections of features that capture specific characteristics of an image. Each group focuses on a different and specific type of visual information. The feature groups used in our experiments are described below:

- Local Binary Patterns (LBP): Local Binary Patterns (LBP) (Ojala *et al.* 1996) is a visual descriptor often used in texture classification. The texture information was extracted by comparing pixel intensity values within a circular neighborhood of a specified radius. The differences were encoded into binary patterns, which were later flattened into a feature vector of size 16384.
- Haralick Features: Using the Gray Level Co-occurrence Matrix (GLCM), Haralick Features (Haralick *et al.* 1973) were computed from texture properties like contrast, dissimilarity, homogeneity, energy, and correlation. These features, with a feature size of 5, provided a detailed analysis of image texture.
- Color Histogram: Color Histogram (Swain and Ballard 1991) was measured from the distribution of pixel intensity values in each Red, Green, and Blue (RGB) color channel. The histograms of these channels were concatenated to form the feature vector of size 768.
- Dominant Colors: Dominant Colors were computed by using k-means clustering. It found the most representative colors (centroids) in an image. The RGB values of these dominant colors form the feature vector of size 9.
- Edge Histogram: Edge Histogram (Stricker and Orengo 1995) was extracted by detecting the edges in the image using the Canny edge detector. It calculated a histogram of the edge intensities with 256 bins, each bin corresponding to an intensity value in the range [0–255]. It formed a feature vector of size 256.
- Histogram of Oriented Gradients (HOG): Histogram of Oriented Gradients (HOG) (Dalal and Triggs 2005) were collected by extracting orientations of the gradients in localized portions of the image, dividing it into cells, and calculating orientation histograms. The “channel_axis” parameter was set to 2, and 256 values were calculated for each channel, resulting in a vector of size 512.
- Scale-Invariant Feature Transform (SIFT): Scale-Invariant Feature Transform (SIFT) (Lowe 2004) detects distinctive key points in the image that are invariant to scale and rotation. The descriptors for these key points were computed and averaged. It resulted in a feature size of 128.
- Oriented FAST and Rotated BRIEF (ORB): Oriented Features from Accelerated Segment Test (FAST) and Rotated Binary Robust Independent Elementary Features (BRIEF), commonly known as ORB, (Rublee *et al.* 2011) is a combination of the FAST key point detector and BRIEF descriptor. It is optimized for computational efficiency and rotation invariance. For this feature group, generated descriptors were measured and averaged to create a feature vector of size 32.
- Pixel Intensity Statistics: Pixel Intensity Statistics were calculated from the means and standard deviations of pixel intensity values for the image. Two arrays containing three means and three standard deviations were generated. By combining them, the feature vector of size 6 was obtained.
- Image Entropy: Image Entropy (Shannon 1948) was measured from the randomness or complexity in the image’s pixel intensity distribution using Shannon entropy. The feature size was 1.
- Discrete Cosine Transform (DCT) Features: Discrete Cosine Transform (DCT) Features (Ahmed *et al.* 1974) were generated by applying the Discrete Cosine Transform (DCT) to the grayscale image. It captured frequency-domain information by encoding spatial patterns. These were flattened to get the feature vector of size 16384.

Recursive Feature Elimination (RFE) is a feature selection technique that selects a subset of all features containing important features by eliminating the less important ones. RFE systematically identifies and retains the most informative features while discarding those with limited predictive value. The algorithm begins by training a wrapper model on the complete feature set and calculating importance scores for each feature. These scores are derived from model-specific metrics such as coefficients in linear models or feature importance in tree-based approaches. Features are then ranked hierarchically according to these scores. Mathematically, given a training dataset with features $X=x_{1},x_{2},\ldots ,x_{n}$, target values *y* and wrapper model *f*, RFE proceeds as follows:

1. Initialize the feature subset S=X
2. For each iteration *t*:
  - Train model *f* using features in *S*: $f_{t}=\text{train}(S,y)$
  - Compute feature importance scores: $w_{t}=\text{importance}(f_{t})$
  - Identify the least important feature: $j=\text{argmin}(w_{t})$
  - Update feature subset: $S=S\setminus x_{j}$
  - Continue until |S| reaches the target feature count

This iterative elimination process yields a sequence of nested feature subsets $S_{1}⊃S_{2}⊃\ldots ⊃S_{k}$, where each subset theoretically contains increasingly informative features. The optimal subset is determined by selecting the subset that maximizes classification accuracy while minimizing dimensionality.

For the feature selection process, 12 different traditional ML models were explored in this study, including k-Nearest Neighbors (**k-NN**) (Peterson 2009), Random Forest (**RF**) (Breiman 2001), Decision Tree (**DT**) (Quinlan 1986), Logistic Regression (**LR**) (Cox 1958), Naive Bayes (**NB**) (Rish *et al.* 2001), Adaptive Boosting (**AdaBoost or AB**) (Freund and Schapire 1997), Light Gradient Boosting Machine (**LightGBM or LGBM**) (Ke *et al.* 2017), Multi-Layer Perceptron **(MLP)** (Rumelhart *et al.* 1986), Linear Discriminant Analysis (**LDA**) (Fisher 1936), Stochastic Gradient Descent Classifier (**SGD**) (Robbins and Monro 1951), Extreme Gradient Boosting (**XGBoost or XGB**) (Chen and Guestrin 2016), and Support Vector Machine (**SVM**) (Cortes 1995). Among these models, one was chosen as the wrapper model to run Recursive Feature Elimination (RFE).

RFE contributes to model robustness. It reduces overfitting by eliminating noise-inducing features and enhances interpretability by focusing on truly impactful variables. It also decreases computational complexity and mitigates the curse of dimensionality. In this study, the Yeo–Johnson transformation (Yeo and Johnson 2000) was applied to the feature groups to make the data distribution resemble Gaussian-like. Five hundred twelve features were selected by running RFE that can be found in Table 6.

### 2.3 Performance metrics

Several well-reputed performance metrics (Altman and Bland 1994, Fawcett 2006, Keilwagen *et al.* 2014, Powers 2020) were utilized in our study for comparison and examination of different models and methodologies. The following metrics were used: Recall (REC), Specificity (SPEC), Accuracy (ACC), Precision (PREC), F1 Score (F1), Matthews Correlation Coefficient (MCC), Area Under the ROC Curve (AUC or AUROC), and Area Under the Precision-Recall Curve (AUPR). The “Macro” version of each metric was employed. The equations for each one of them are provided below:

#### 2.3.1 Recall

Recall measures the model’s ability to correctly identify positive samples of each class. The formula calculates recall for each class *i* and then averages it over all *C* classes.


(1) $$\text{REC}=\frac{1}{C}\sum_{i=1}^{C}{\text{REC}}_{i}=\frac{1}{C}\sum_{i=1}^{C}\frac{TP_{i}}{TP_{i}+FN_{i}}$$

Here,

-
$TP_{i}$ (True Positive): The number of correct predictions for class *i*
-
$FN_{i}$ (False Negative): The number of actual samples of class *i* that were predicted as some other class

#### 2.3.2 Specificity

Specificity measures the model’s ability to correctly identify negative samples of each class. It is calculated for each class *i* and then averaged over all *C* classes.


(2) $$\text{SPEC}=\frac{1}{C}\sum_{i=1}^{C}{\text{SPEC}}_{i}=\frac{1}{C}\sum_{i=1}^{C}\frac{TN_{i}}{TN_{i}+FP_{i}}$$

Here,

-
$TN_{i}$ (True Negative): The number of correct predictions for class *i* when the sample is not of class *i*
-
$FP_{i}$ (False Positive): The number of samples predicted as class *i* when they belong to other classes

#### 2.3.3 Accuracy

Accuracy is the proportion of all correctly predicted samples (true positives + true negatives) to the total samples in the dataset. This is calculated for each class *i* and then averaged over all *C* classes.


(3) $$\text{ACC}=\frac{1}{C}\sum_{i=1}^{C}{\text{ACC}}_{i}=\frac{1}{C}\sum_{i=1}^{C}\frac{TP_{i}+TN_{i}}{TP_{i}+TN_{i}+FP_{i}+FN_{i}}$$

Here,

-
$TP_{i}$ + $TN_{i}$: Correct predictions (both positive and negative) for class *i*
-
$FP_{i}$ + $FN_{i}$: Incorrect predictions for class *i*

#### 2.3.4 Precision

Precision measures the ability of the model to avoid false positives. It is calculated for each class *i* and then averaged over all *C* classes.


(4) $$\text{PREC}=\frac{1}{C}\sum_{i=1}^{C}{\text{PREC}}_{i}=\frac{1}{C}\sum_{i=1}^{C}\frac{TP_{i}}{TP_{i}+FP_{i}}$$

Here,

-
$TP_{i}$ (True Positive): The correct predictions for class i
-
$FP_{i}$ (False Positive): The incorrect predictions for class i

#### 2.3.5 F1 score

F1 Score is the harmonic mean of Precision and Recall, balancing the two. It is calculated for each class *i* and then averaged over all *C* classes.


(5) $$F1=\frac{1}{C}\sum_{i=1}^{C}{F1}_{i}=\frac{1}{C}\sum_{i=1}^{C}\frac{2\cdot {\text{PREC}}_{i}\cdot {\text{REC}}_{i}}{{\text{PREC}}_{i}+{\text{REC}}_{i}}$$

Here,

- PREC${\;}_{i}$: Precision for class *i*
- REC${\;}_{i}$: Recall for class *i*

#### 2.3.6 Matthews correlation coefficient

MCC is a measure of the quality of the classifications. It is calculated for each class *i* and then averaged over all *C* classes.


(6) $$\begin{array}{c} \text{MCC}=\frac{1}{C}\sum_{i=1}^{C}{\text{MCC}}_{i}\\ =\frac{1}{C}\sum_{i=1}^{C}\frac{TP_{i}\cdot TN_{i}-FP_{i}\cdot FN_{i}}{\sqrt{\left(TP_{i}+FP_{i})(TP_{i}+FN_{i})(TN_{i}+FP_{i})(TN_{i}+FN_{i}\right)}} \end{array}$$

Here, $TP_{i}$, $FP_{i}$, $TN_{i}$ and $FN_{i}$ are similar as stated previously.

#### 2.3.7 Area under the ROC curve

AUC measures the ability of the model to discriminate between classes. It is calculated for each class *i* and then averaged over all *C* classes.


(7) $$\text{AUC}=\frac{1}{C}\sum_{i=1}^{C}{\text{AUC}}_{i}$$

Here,

- AUC${\;}_{i}$: AUC for class *i*

#### 2.3.8 Area under the precision-recall curve

This metric is particularly useful in cases of class imbalance, as it focuses more on the performance of the positive class, rather than considering all classes equally as in AUC. It is calculated for each class *i* and then averaged over all *C* classes.


(8) $$\text{AUPR}=\frac{1}{C}\sum_{i=1}^{C}{\text{AUPR}}_{i}$$

Here,

- AUPR${\;}_{i}$: AUPR for class *i*

### 2.4 Image augmentation

The pipeline for generating new images was designed to enhance the diversity of training data by applying the following series of transformations to the existing images:

- Random Horizontal Flip: It horizontally flipped the images with a probability of 50%.
- Random Rotation: It rotated the images randomly within a range of ±30°.
- Color Jittering: It randomly adjusted the brightness, contrast, saturation, and hue of images up to 50% for each.
- Random Resized Crop: Firstly, it extracted a crop from the images with a random size in the range of 80% to 100% of the original image area. After that, it resized the resultant images back to 128 × 128 resolution.
- Random Affine Transformations: It applied affine transformations with random rotation of up to ±30° and also added a shear of up to 10°.
- Noise Injection: Lastly, Gaussian noise was injected into the images.

The genetic algorithm to select images for image augmentation is provided in Algorithm 1. Firstly, new images were generated from the D3 (Training + Validation) set by applying our pipeline. Initially, a population was created having *N* random individuals. Each individual had *S* number of image samples. In each generation, the fitness score of each individual belonging to the population was calculated. Then, the individuals were sorted based on their fitness scores, and the top *T* individuals were taken. A new population was created for the next generation, and these *T* individuals were added to it. The remaining required individuals in the new population were generated by mutating the top *T* individuals. During the mutation process, a parent was randomly chosen from the top *T* individuals, and its existing samples were replaced with other samples in the whole population with a mutation rate *r*. This new population was used in the next generation. After conducting the genetic algorithm generation by generation, the best individual was selected based on the fitness scores from the population of the last generation. This was augmented with the original dataset and used in our final model.

**Figure 2:**
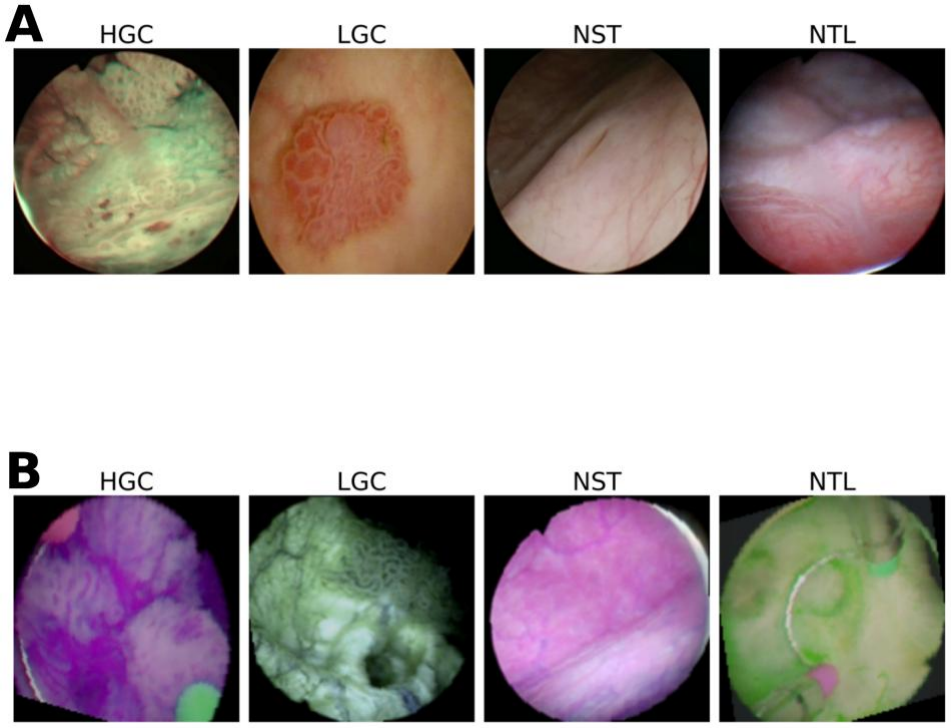
Visualization of representative samples from the original and augmented datasets. (A) Illustrations of some sample images from the original dataset with resolution resized to 224 × 224. (B) Illustrations of some sample images from the D1 dataset with resolution resized to 224 × 224. Images from each class (HGC, LGC, NST, and NTL) were randomly selected from the respective datasets.

The fitness scores inside the genetic algorithm were measured from the accuracy scores given by a traditional ML classifier. To select the suitable classifier, 10-fold cross-validation (CV) of the traditional 12 ML models (as listed in Section 2.2) was conducted. Images were flattened and fed to these 12 ML models. The images were resized to 128 × 128 dimensions and normalized before input. For similar reasons mentioned in Section 2.2, 224 × 224 dimensions were avoided. Light Gradient Boosting Machine (LGBM) was chosen as the most suitable model for conducting the genetic algorithm-based image augmentation. Five hundred new samples were selected for image augmentation. The visuals of our existing dataset and new augmented D1 set are illustrated in Fig. 2. The LGBM model was configured with 100 estimators (n_estimators = 100) and a maximum tree depth of 3 (max_depth = 3), while all other parameters were kept at their default values.

Algorithm 1 Genetic Algorithm to Select Images for Image Augmentation

1: **Input:** Existing images $E_{A}$, labels of the existing images $E_{L}$, model *M*, number of generations *G*, number of individuals *N*, image sample size per individual *S*, number of top individuals to retain *T*

2: **Output:** Selected top augmented images and their labels

3: Generate images for augmentation A from $E_{A}$ and their corresponding labels L from $E_{L}$

4: Initialize population P with *N* random individuals, each containing *S* number of image samples from A

5: **for**  g=1 to *G* **do** 

6: Calculate fitness for each individual in P:

7: **for** each individual *I* in P  **do** 

8: Let $A_{I}\subseteq A$ be the images of *I* and $L_{I}\subseteq L$ be their labels

9: Compute accuracy fitness (*I*) ← evaluate_model $\left(A_{I},L_{I},M\right)$

10: **end for** 

11: Sort the individuals in P by accuracy fitness scores and select top *T* individuals to form T

12: Create population of next generation $P^{\prime }$ and add all individuals from T to $P^{\prime }$

13: **while**  $|P^{\prime }|<N$  **do** 

14: Select a parent *Pr* from T randomly

15: Generate a child *Ch* by mutating *Pr* with mutation rate *r*

16: Number of mutations m←⌊r·|Pr|⌋

17: **for**  i=1 to *m* **do** 

18: Select a random index j∈{1,…,|Pr|}

19: Replace *Ch[j]* with a random index k∈{1,…,|A|}

20: **end for** 

21: Add *Ch* to $P^{\prime }$

22: **end while** 

23: Update $P\leftarrow P^{\prime }$

24: **end for** 

25: Select the best individual $I^{*}$ from the population P of the final generation

26: Return images $A_{I^{*}}$ and their labels $L_{I^{*}}$

### 2.5 Model selection

The timm library (Wightman 2019) was utilized to run the models. The evaluated models included the pretrained (on the ImageNet dataset) models of: **MobileNetV3** (tf_mobilenetv3_large_minimal_100.in1k) (Howard *et al.* 2019), **Resnet-50** (resnet50.a1_in1k) (He *et al.* 2016), **DenseNet** (densenet201.tv_in1k) (Huang *et al.* 2017), **EfficientFormer-V2** (efficientformerv2_l.snap_dist_in1k) (Li *et al.* 2022), **Twins-PCPVT** (twins_pcpvt_base.in1k) (Chu *et al.* 2021), **CoAtNet** (coatnet_0_rw_224.sw_in1k) (Dai *et al.* 2021), **Swin Transformer** (swin_base_patch4_window7_224.ms_in22k_ft_in1k) (Liu *et al.* 2021), **Swin Transformer V2** (swinv2_tiny_window8_256.ms_in1k) (Liu *et al.* 2022a), **Inception-v3** (inception_v3.tv_in1k) (Szegedy *et al.* 2016), and **ConvNeXt** (convnext_base.fb_in22k_ft_in1k) (Liu *et al.* 2022b). Each one of these models was fed images as input after resizing them to a 224 × 224 resolution and normalizing them. These models were analyzed inside the CNN branch of our proposed model architecture. From the validation results, MobileNetV3 was selected among these models.

### 2.6 Framework

The framework of our proposed model is provided in Fig. 4. The architecture was divided into two branches. One was the FNN branch, and the other was the CNN branch. The descriptions of each of the branches are provided below:

The inputs to the FNN branch were the handcrafted features that were extracted and then went through the feature selection process of RFE. Two linear layers were present in this branch. The first linear layer had input and output sizes of 512 and 256, respectively. The second linear layer had sizes of 256 and 128 as its input and output, respectively.

**Figure 3:**
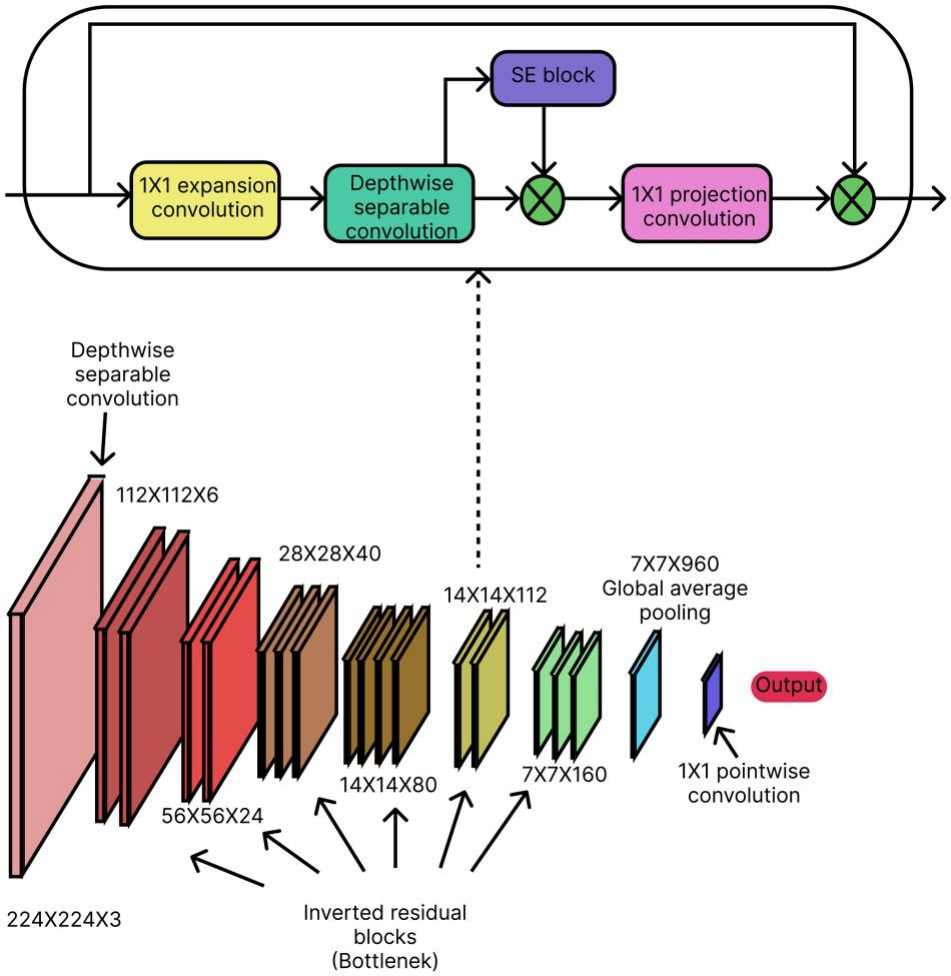
The architecture of the MobileNetV3 model. The input sizes are mentioned for each of the block segments. The model processes a 224 × 224 × 3 input image through a series of inverted residual (bottleneck) blocks consisting of 1 × 1 expansion convolutions, depthwise separable convolutions, squeeze-and-excitation (SE) blocks, and 1 × 1 projection convolutions. The feature maps are progressively reduced from 112 × 112 × 6 to 7 × 7 × 960, followed by global average pooling, a 1 × 1 pointwise convolution, and the output layer.

**Figure 4:**
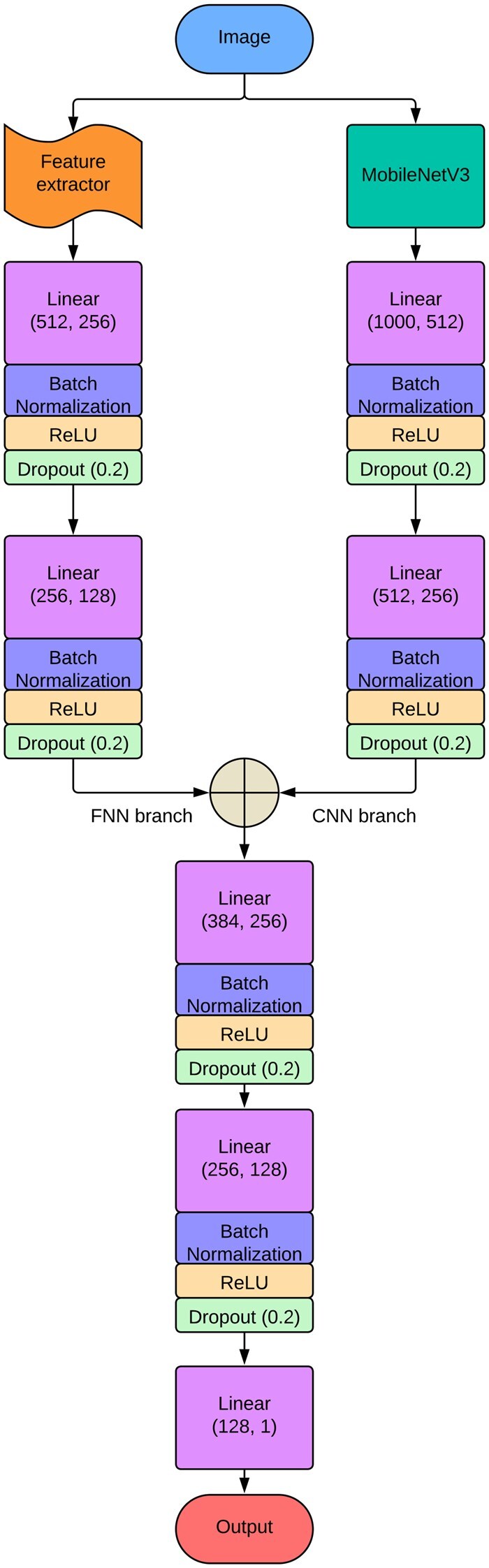
Architectural overview of the proposed DeepBCTPred hybrid model. The framework consists of two parallel branches: a CNN branch, where MobileNetV3 is used as a deep feature extractor followed by fully connected layers with batch normalization, ReLU activations, and dropout; and an FNN branch, which processes the selected handcrafted features through multiple fully connected layers. The outputs from both branches are fused and passed through additional fully connected layers to perform the final classification. Here, Linear (X, Y) represents a fully connected (dense) layer that transforms input of size X into output of size Y, and Dropout (p) represents a dropout layer having drop rate p.

The input to the CNN branch was the sample image. MobileNetV3 (Howard *et al.* 2019) was used inside this branch. To improve feature representation, MobileNetV3 combines Squeeze-and-Excitation (SE) (Howard *et al.* 2019) modules, inverted residual blocks with linear bottlenecks [from MobileNetV2 (Sandler *et al.* 2018)], and depthwise separable convolutions. To increase computational efficiency, it makes use of a hard-swish (h-swish) (Howard *et al.* 2019) activation in a Neural Architecture Search (NAS)-optimized structure. A lightweight stem, several inverted residual blocks with different expansion ratios, SE attention mechanisms, and an optimal final classification head make up the network. The architecture of the MobileNetV3 model is shown in Fig. 3. Before depthwise separable convolutions, which lower computational costs by factorizing standard convolutions into depthwise and pointwise (1×1) convolutions, the architecture starts with a 3×3 standard convolution. It makes use of inverted residual blocks with linear bottlenecks, in which the input channels are expanded before a linear projection, and a depthwise convolution is applied. Squeeze-and-Excitation (SE) modules are incorporated into select blocks to dynamically recalibrate feature importance. In order to improve non-linearity while preserving efficiency, the model substitutes hard-swish (h-swish) activation for ReLU activation in key layers. The last layers consist of a 1×1 convolution, global average pooling, and a fully connected (FC) layer for classification. In Fig. 4, MobileNetV3 output a tensor of size (Batch_size, 1000). This output was passed as input to the next linear layer with a 1000 input size and a 512 output size. After that, another linear layer was added that had input and output sizes of 512 and 256, respectively.

The outputs of both branches are then concatenated and fed to a linear layer (384, 256), where 384 is the input size, and 256 is the output size. Another linear layer (256, 128) was added after that. Finally, a linear layer was used in the output layer of our architecture.

Every linear layer except the last one was followed by batch normalization, ReLU activation, and dropout with a rate of 0.2. Batch normalization and dropout were used to improve generalization and convergence. ReLU was used to bring non-linearity to the model.

The model configurations of our proposed model, DeepBCTPred, are given in Table 4.

**Table 4:** Model configuration of the proposed model DeepBCTPred.

Name of the parameters	Values
Input image resolution	(224 × 224)
Input feature size	512
Activation Function (hidden layer)	ReLU
Activation Function (output layer)	Sigmoid
Loss function	Cross-entropy loss
Optimizer	Adam
Initial learning rate	0.0001
Epoch	96
Dropout	0.2
Batch size	32
Batch normalization	1D

## 3 Results

In this section, the results of different experiments are depicted. Firstly, the ML model selection for running RFE, and then the feature selection results are shown. Then, the model selection for running the genetic algorithm for image augmentation is discussed. After the image augmentation, validation results and comparison with the SOTA methods are described. Different plots are also analyzed.

### 3.1 Selection of model used for RFE feature selection

Ten-fold CV was conducted using the traditional 12 ML models (as listed in Section 2.2) on the D3 (Training + Validation) set. The models were trained using the concatenated 11 feature groups (as listed in Section 2.2). The 10-fold CV performance results are shown in Table 5. Here, LGBM outperformed all other models. So, LGBM was selected as the wrapper model to run RFE. The summary of the selected features after running RFE is provided in Table 6. The total number of extracted features across all feature groups is 34 485, of which 512 features were selected after applying RFE.

**Table 5:** Ten-fold CV performance of 12 ML models on the D3 set, using the concatenated 11 feature groups.^a^

Model	REC	SPEC	ACC	PREC	F1	MCC	AUC	AUPR
KNN	0.4896 ± 0.0253	0.8376 ± 0.0086	0.4896 ± 0.0253	0.7407 ± 0.0170	0.4728 ± 0.0342	0.4156 ± 0.0334	0.7894 ± 0.0157	0.5869 ± 0.0217
RF	0.7406 ± 0.0299	0.9521 ± 0.0081	0.7406 ± 0.0299	0.9066 ± 0.0155	0.7512 ± 0.0389	0.7415 ± 0.0388	0.9787 ± 0.0060	0.9265 ± 0.0137
DT	0.7518 ± 0.0502	0.9291 ± 0.0131	0.7518 ± 0.0502	0.7521 ± 0.0531	0.7476 ± 0.0494	0.6800 ± 0.0619	0.8290 ± 0.0447	0.6340 ± 0.0639
LR	0.8023 ± 0.0292	0.9548 ± 0.0075	0.8023 ± 0.0292	0.8786 ± 0.0352	0.8230 ± 0.0309	0.7892 ± 0.0362	0.9723 ± 0.0094	0.9117 ± 0.0222
NB	0.5598 ± 0.0581	0.8528 ± 0.0163	0.5598 ± 0.0581	0.6027 ± 0.0396	0.4976 ± 0.0472	0.4123 ± 0.0611	0.7308 ± 0.0334	0.4531 ± 0.0356
AB	0.6493 ± 0.0407	0.8855 ± 0.0112	0.6493 ± 0.0407	0.6640 ± 0.0421	0.6527 ± 0.0403	0.5410 ± 0.0500	0.8727 ± 0.0263	0.6626 ± 0.0562
LGBM	**0.8692 **± 0.0263	**0.9768 **± 0.0042	**0.8692 **± 0.0263	**0.9369 **± 0.0164	**0.8905 **± 0.0242	**0.8760 **± 0.0217	**0.9938 **± 0.0030	**0.9716 **± 0.0111
MLP	0.7982 ± 0.0382	0.9486 ± 0.0113	0.7982 ± 0.0382	0.8281 ± 0.0506	0.8068 ± 0.0426	0.7599 ± 0.0527	0.9638 ± 0.0095	0.8817 ± 0.0317
LDA	0.4691 ± 0.1686	0.8286 ± 0.0593	0.4691 ± 0.1686	0.6067 ± 0.1134	0.4486 ± 0.1925	0.3413 ± 0.2089	0.7079 ± 0.1388	0.5510 ± 0.1661
SGD	0.7968 ± 0.0211	0.9472 ± 0.0073	0.7968 ± 0.0211	0.8187 ± 0.0387	0.8030 ± 0.0263	0.7538 ± 0.0334	0.8846 ± 0.0196	0.6619 ± 0.0325
XGB	0.8524 ± 0.0282	0.9739 ± 0.0060	0.8524 ± 0.0282	0.9338 ± 0.0211	0.8755 ± 0.0252	0.8611 ± 0.0254	0.9926 ± 0.0020	0.9626 ± 0.0101
SVM	0.7202 ± 0.0284	0.9418 ± 0.0085	0.7202 ± 0.0284	0.8817 ± 0.0165	0.7334 ± 0.0369	0.7116 ± 0.0355	0.9638 ± 0.0123	0.8689 ± 0.0365

a The means and standard deviations were calculated from the 10-fold results. The values are reported in “mean ± std_dev” format. The highest scores for each metric are shown in bold.

**Table 6:** Summary of the selected features after the feature selection of RFE.

Feature group	Total feature size	Number of selected features
LBP	16 384	35
Haralick features	5	4
Color histogram	768	54
Dominant colors	9	1
Edge histogram	256	2
HOG	512	123
SIFT	128	36
ORB	32	8
Pixel intensity statistics	6	5
Image entropy	1	1
DCT features	16384	243

### 3.2 Selection of model to run the genetic algorithm for image augmentation

10-Fold CV was done using the traditional 12 ML models (as listed in Section 2.2) on the D3 set for choosing the model to run the genetic algorithm. The models were trained using the flattened image representations. The 10-Fold CV performance results are provided in Table 7. Here, LGBM performed the best in almost all metrics. So, LGBM was selected to run the genetic algorithm.

**Table 7:** Ten-fold CV performance of 12 ML models on the D3 set, utilizing flattened image representations.^a^

Model	REC	SPEC	ACC	PREC	F1	MCC	AUC	AUPR
KNN	0.8572 ± 0.0541	0.9642 ± 0.0098	0.8572 ± 0.0541	0.9052 ± 0.0282	0.8726 ± 0.0454	0.8427 ± 0.0495	0.9784 ± 0.0098	0.9365 ± 0.0211
RF	0.8843 ± 0.0326	0.9697 ± 0.0084	0.8843 ± 0.0326	0.9290 ± 0.0185	0.9005 ± 0.0279	0.8747 ± 0.0313	0.9890 ± 0.0069	0.9675 ± 0.0166
DT	0.7569 ± 0.0438	0.9266 ± 0.0080	0.7569 ± 0.0438	0.7525 ± 0.0421	0.7523 ± 0.0427	0.6805 ± 0.0487	0.8285 ± 0.0321	0.6311 ± 0.0465
LR	0.8529 ± 0.0293	0.9599 ± 0.0078	0.8529 ± 0.0293	0.8870 ± 0.0301	0.8654 ± 0.0276	0.8289 ± 0.0338	0.9695 ± 0.0055	0.9181 ± 0.0271
NB	0.6012 ± 0.0320	0.8530 ± 0.0125	0.6012 ± 0.0320	0.5685 ± 0.0383	0.5112 ± 0.0330	0.4177 ± 0.0444	0.7347 ± 0.0253	0.4345 ± 0.0322
AB	0.6181 ± 0.0427	0.8797 ± 0.0145	0.6181 ± 0.0427	0.6453 ± 0.0620	0.6206 ± 0.0456	0.5078 ± 0.0569	0.8480 ± 0.0172	0.5860 ± 0.0325
LGBM	**0.9039** ± 0.0250	**0.9763** ± 0.0056	**0.9039** ± 0.0250	0.9327 ± 0.0172	**0.9154** ± 0.0213	**0.8942** ± 0.0240	**0.9921** ± 0.0030	0.9718 ± 0.0092
MLP	0.8633 ± 0.0519	0.9644 ± 0.0098	0.8633 ± 0.0519	0.8860 ± 0.0586	0.8714 ± 0.0552	0.8387 ± 0.0634	0.9746 ± 0.0067	0.9253 ± 0.0331
LDA	0.8139 ± 0.0391	0.9486 ± 0.0054	0.8139 ± 0.0391	0.8374 ± 0.0356	0.8194 ± 0.0339	0.7723 ± 0.0362	0.9567 ± 0.0121	0.8807 ± 0.0232
SGD	0.8235 ± 0.0393	0.9496 ± 0.0116	0.8235 ± 0.0393	0.8545 ± 0.0414	0.8321 ± 0.0364	0.7875 ± 0.0462	0.8809 ± 0.0328	0.6738 ± 0.0774
XGB	0.8976 ± 0.0275	0.9744 ± 0.0079	0.8976 ± 0.0275	**0.9332** ± 0.0179	0.9111 ± 0.0217	0.8888 ± 0.0275	0.9907 ± 0.0061	**0.9739** ± 0.0104
SVM	0.7910 ± 0.0245	0.9543 ± 0.0061	0.7910 ± 0.0245	0.9070 ± 0.0143	0.8182 ± 0.0262	0.7919 ± 0.0270	0.9619 ± 0.0109	0.9116 ± 0.0277

a The means and standard deviations were calculated from the 10-fold results. The values are reported in “mean ± std_dev” format. The highest scores for each metric are shown in bold.

### 3.3 Validation results to select the CNN branch of the final model

**Figure 5:**
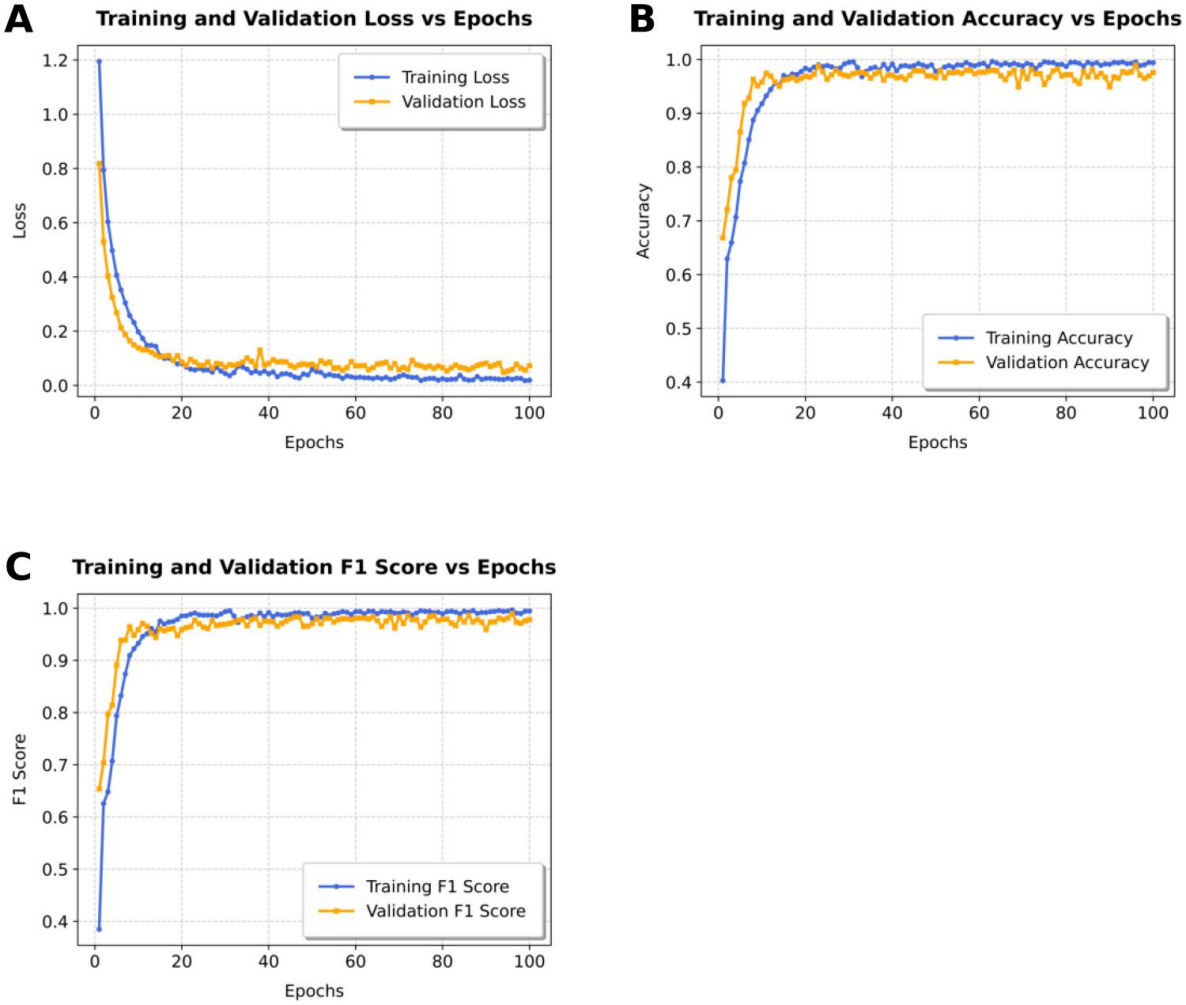
Learning curve plots during training and validation. (A) Training and validation loss vs. epochs. (B) Training and validation accuracy vs. epochs. (C) Training and validation F1 score vs. epochs.

Different CNN-based deep learning models were analyzed inside the CNN branch of our model architecture. The models were trained on the D2 (Training + Augmentation) dataset and tested on the validation set. Ten different models (as listed in Section 2.5) were explored inside the CNN branch, and their scores are reported in Table 8. The full validation results are available at Supplementary file, available as supplementary data at *Bioinformatics Advances* online. The model that had MobileNetV3 in its CNN branch outperformed all other models based on all metrics. So, this was chosen to be included in our final model, named **DeepBCTPred**. Three different plots of the model containing MobileNetV3 are provided in Fig. 5. The three plots included: Training and Validation Loss vs. Epochs, Training and Validation Accuracy vs. Epochs, and Training and Validation F1 score vs. Epochs.

**Table 8:** Performance of different models trained on the D2 dataset and tested on the validation set. The highest scores for each metric are shown in bold.

Models inside CNN branch	REC	SP	ACC	PREC	F1	MCC	AUC	AUPR
MobileNetV3	**0.9898**	**0.9953**	**0.9898**	**0.99**	**0.9899**	**0.9852**	**0.999**	**0.9981**
Resnet-50	0.9808	0.9919	0.9808	0.9758	0.978	0.9703	0.9988	0.9972
DenseNet	0.9679	0.9932	0.9679	0.9843	0.9755	0.9695	0.9966	0.9885
EfficientFormer-V2	0.9745	0.9926	0.9745	0.9672	0.9706	0.9631	0.9984	0.9925
Twins-PCPVT	0.9741	0.9887	0.9741	0.9668	0.9703	0.9589	0.9979	0.9923
CoAtNet	0.9721	0.9874	0.9721	0.9647	0.9683	0.9557	0.9983	0.9966
Swin Transformer	0.9562	0.9871	0.9562	0.9729	0.964	0.9517	0.9969	0.9924
Swin Transformer V2	0.9507	0.9886	0.9507	0.9736	0.9611	0.9507	0.998	0.9925
Inception-v3	0.9594	0.9893	0.9594	0.958	0.9584	0.9477	0.9947	0.9812
ConvNeXt	0.9552	0.9828	0.9552	0.9427	0.9479	0.9309	0.9965	0.9889

### 3.4 Comparison with the SOTA methods on the test set

The proposed model, DeepBCTPred, was compared with several state-of-the-art (SOTA) methods, including 17DCNN (Devi *et al.* 2024), AlexNet (Sunnetci *et al.* 2025), ResNet_18 + SVM (Sunnetci *et al.* 2025), ViT (Sunnetci *et al.* 2025), CNN (Lutviana *et al.* 2025), and ResNet50 (Sathya *et al.* 2025), as presented in Table 9. The original models or source codes for these methods were not made publicly available. Attempts were made to contact the respective authors—Lazo *et al.* 2023, Tiwari *et al.* 2023, Devi *et al.* 2024, Sunnetci *et al.* 2025, Lutviana *et al.* 2025, Sathya *et al.* 2025—via email to obtain the necessary models or code, but no responses were received. Consequently, only the models proposed by Sunnetci *et al.* (2025), Devi *et al.* (2024), Lutviana *et al.* (2025), and Sathya *et al.* (2025) were reproduced using the architectural and configuration details provided in their respective publications. The remaining models could not be reproduced. All models were trained on the D4 (Training + Validation + Augmentation) dataset and evaluated on the test set.

**Table 9:** Comparison of all SOTA methods on the test set.^a^

Model	REC	SP	ACC	PREC	F1	MCC	AUC	AUPR
Devi *et al.* (2024) (17DCNN)	0.8593	0.9724	0.8593	0.8748	0.8658	0.8402	0.9794	0.9263
Sunnetci *et al.* (2025) (AlexNet)	0.8984	0.9815	0.8984	0.9406	0.9158	0.9007	0.9973	0.9755
Sunnetci *et al.* (2025) (ResNet_18 + SVM)	0.8866	0.9771	0.8866	0.8948	0.8903	0.8677	0.9944	0.9749
Sunnetci *et al.* (2025) (ViT)	0.9065	0.9786	0.9065	0.9166	0.911	0.89	0.9782	0.9528
Lutviana *et al.* (2025) (CNN)	0.8967	0.9748	0.8967	0.9322	0.9118	0.8900	0.9937	0.9762
Sathya *et al.* (2025) (ResNet50)	0.9682	0.9922	0.9682	0.9672	0.9677	0.9598	0.9989	0.9908
DeepBCTPred	**0.9874**	**0.9945**	**0.9874**	**0.9725**	**0.9796**	**0.9738**	**0.9992**	**0.9961**

a All models were trained on the D4 dataset. The highest scores for each metric are shown in bold.

**Figure 6:**
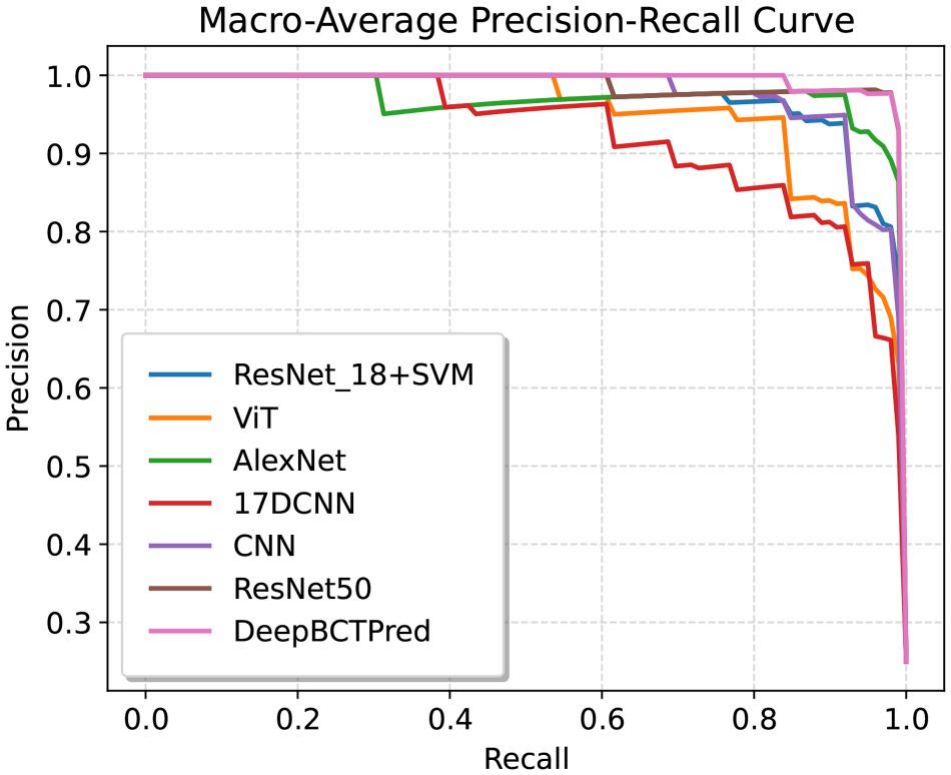
Macro-average precision-recall comparison on the test set among models trained on the D4 dataset. The curve was interpolated from individual class-specific precision-recall curves.

**Figure 7:**
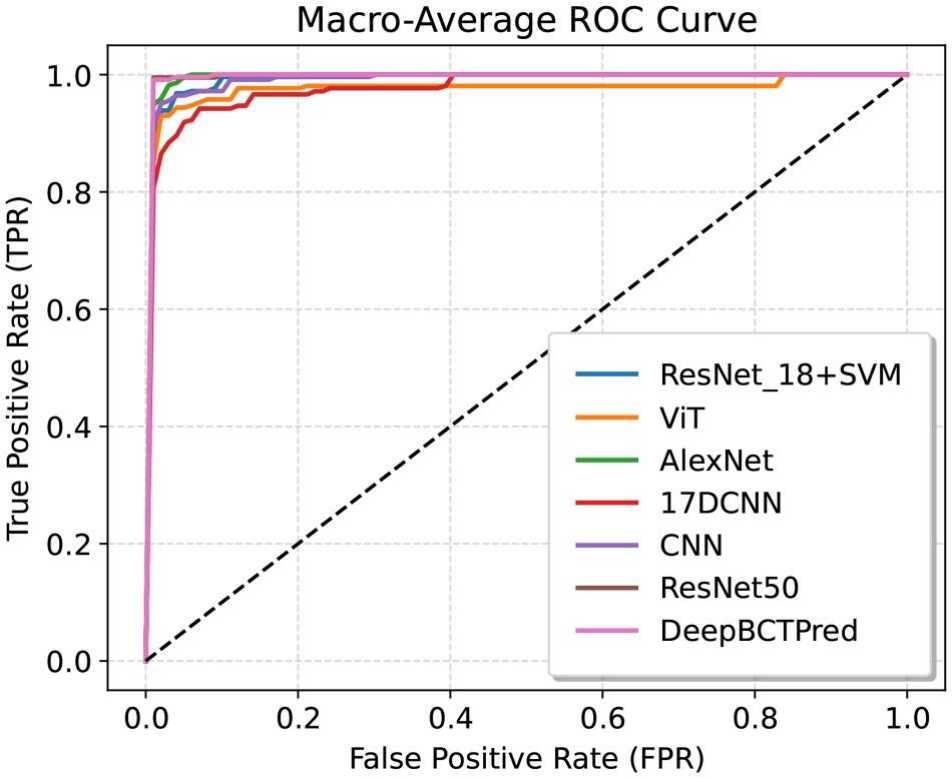
Macro-average ROC comparison on the test set among models trained on the D4 dataset. The curve was interpolated from individual class-specific ROC curves.

DeepBCTPred outperformed the SOTA methods based on all metrics. The interpolated Precision-Recall (PR) curve and Receiver Operating Characteristic (ROC) curve are provided in Figs 6 and 7, respectively. Firstly, the PR curves and ROC curves of each class were generated, and from them, the macro average PR curve and ROC curve were interpolated. It can be seen that DeepBCTPred outperformed the SOTA models.

**Figure 8:**
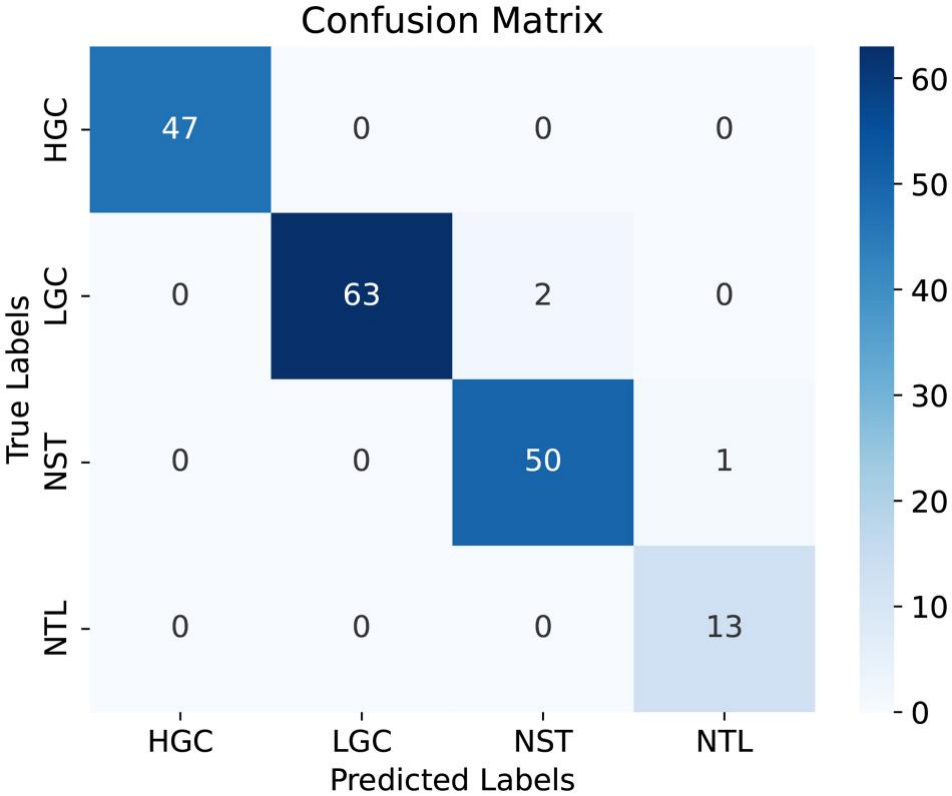
Confusion matrix generated by DeepBCTPred, trained on the D4 dataset and tested on the test set.

**Figure 9:**
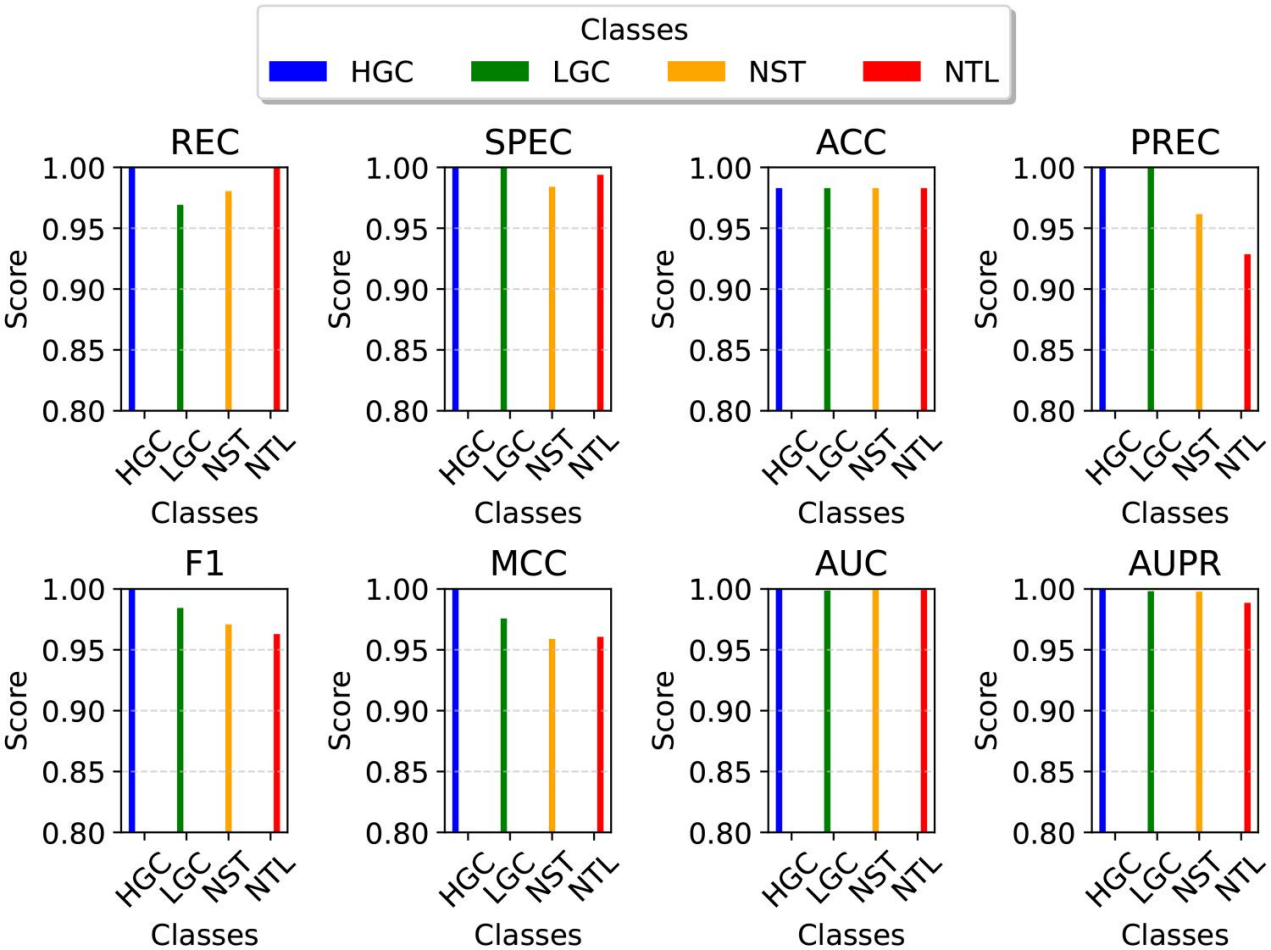
Bar plots representing the class-wise performance metrics of DeepBCTPred, trained on the D4 dataset and evaluated on the test set. To improve visualization, the displayed scores were kept within the range of 0.8 to 1.

The confusion matrix generated by DeepBCTPred, trained on the D4 set and tested on the test set is provided in Fig. 8. The bar plots that represent the class-wise performance metrics of DeepBCTPred, trained on the D4 dataset and evaluated on the test set, are given in Fig. 9. From the figure, it can be said that DeepBCTPred performed consistently across all classes.

**Figure 10:**
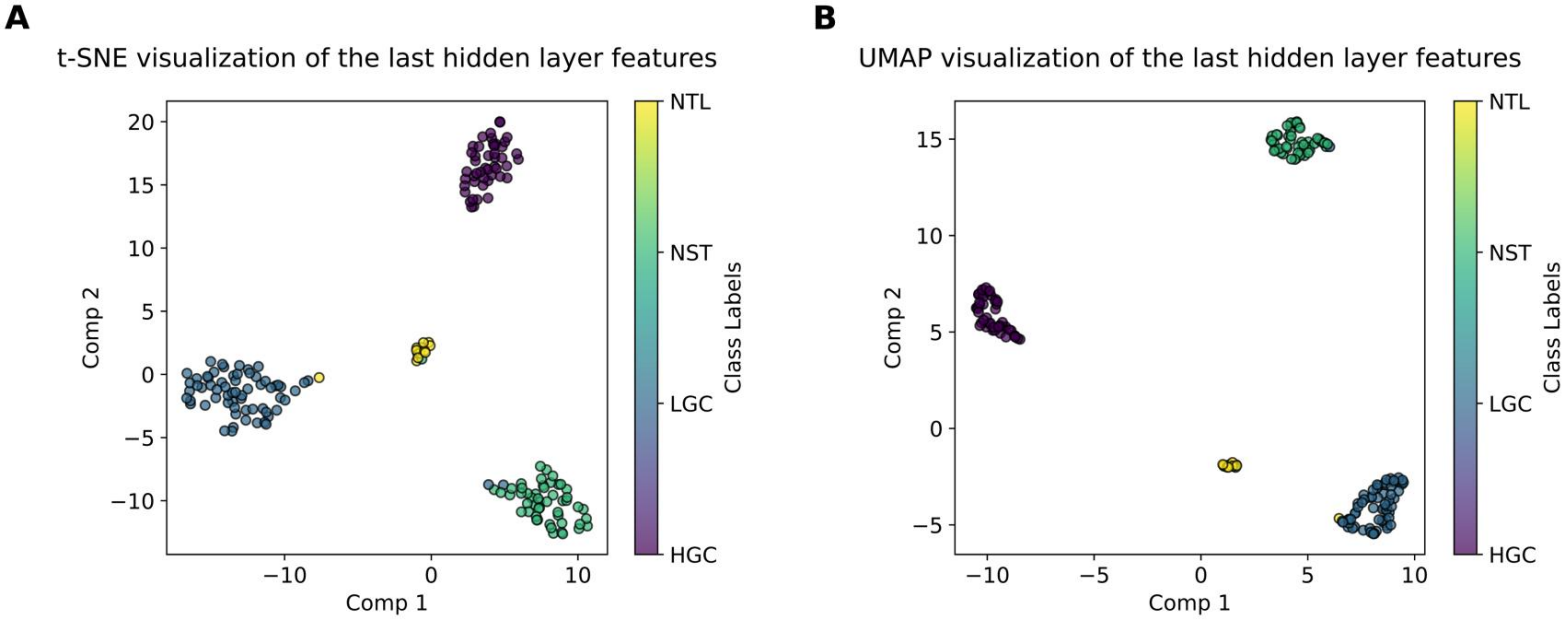
t-SNE and UMAP visualizations of the last hidden layer representations on the test set. (A) t-SNE visualization of the last hidden layer representations on the test set. (B) UMAP visualization of the last hidden layer representations on the test set.

Two different plots: t-distributed Stochastic Neighbor Embedding (t-SNE) (Van der Maaten and Hinton 2008) and Uniform Manifold Approximation and Projection (UMAP) (McInnes *et al.* 2018) are illustrated in Fig. 10. The plots were generated by taking the last hidden layer representation of DeepBCTPred. It can be clearly seen that DeepBCTPred is separating the different class samples. The class separation of DeepBCTPred proved its superiority and effectiveness in bladder cancer tissue prediction.

The performance of three model variations—DeepBCTPred (only FNN branch), DeepBCTPred (only CNN branch), and the full DeepBCTPred model—was compared in an ablation study. Table 10 presents the results, which further confirm the effectiveness of the proposed DeepBCTPred model. DeepBCTPred outperforms individual branches, which highlights the complementary strengths of both the FNN and CNN branches. The performance of two model variations—DeepBCTPred D3 (DeepBCTPred model trained on the D3 set) and the full DeepBCTPred model trained on the D4 set—was compared in another ablation study. Table 11 presents the results, which further confirm the effectiveness of the proposed DeepBCTPred model. Since the D3 set did not include the augmented images from the D1 set, the results emphasize the benefit of incorporating augmented images during training, demonstrating their impact on model performance.

**Table 10:** Ablation study results: performance comparison of DeepBCTPred (only FNN branch), DeepBCTPred (only CNN branch), and the full DeepBCTPred model.^a^

Model	REC	SP	ACC	PREC	F1	MCC	AUC	AUPR
DeepBCTPred (only FNN branch)	0.8979	0.9798	0.8979	0.9536	0.9181	0.9031	0.984	0.9473
DeepBCTPred (only CNN branch)	0.9489	0.9905	0.9489	0.9598	0.9538	0.9447	0.9991	0.9893
DeepBCTPred	**0.9874**	**0.9945**	**0.9874**	**0.9725**	**0.9796**	**0.9738**	**0.9992**	**0.9961**

a Models were trained on the D4 set and evaluated on the test set. The highest scores for each metric are shown in bold.

**Table 11:** Ablation study results: performance comparison of DeepBCTPred D3 (DeepBCTPred model trained on the D3 set) and the full DeepBCTPred model trained on the D4 set.^a^

Model	REC	SP	ACC	PREC	F1	MCC	AUC	AUPR
DeepBCTPred D3	0.9528	0.9926	0.9528	0.9641	0.958	0.9509	0.9985	0.9779
DeepBCTPred	**0.9874**	**0.9945**	**0.9874**	**0.9725**	**0.9796**	**0.9738**	**0.9992**	**0.9961**

a Models were evaluated on the test set. The highest scores for each metric are shown in bold.

### 3.5 Model interpretation

**Figure 11:**
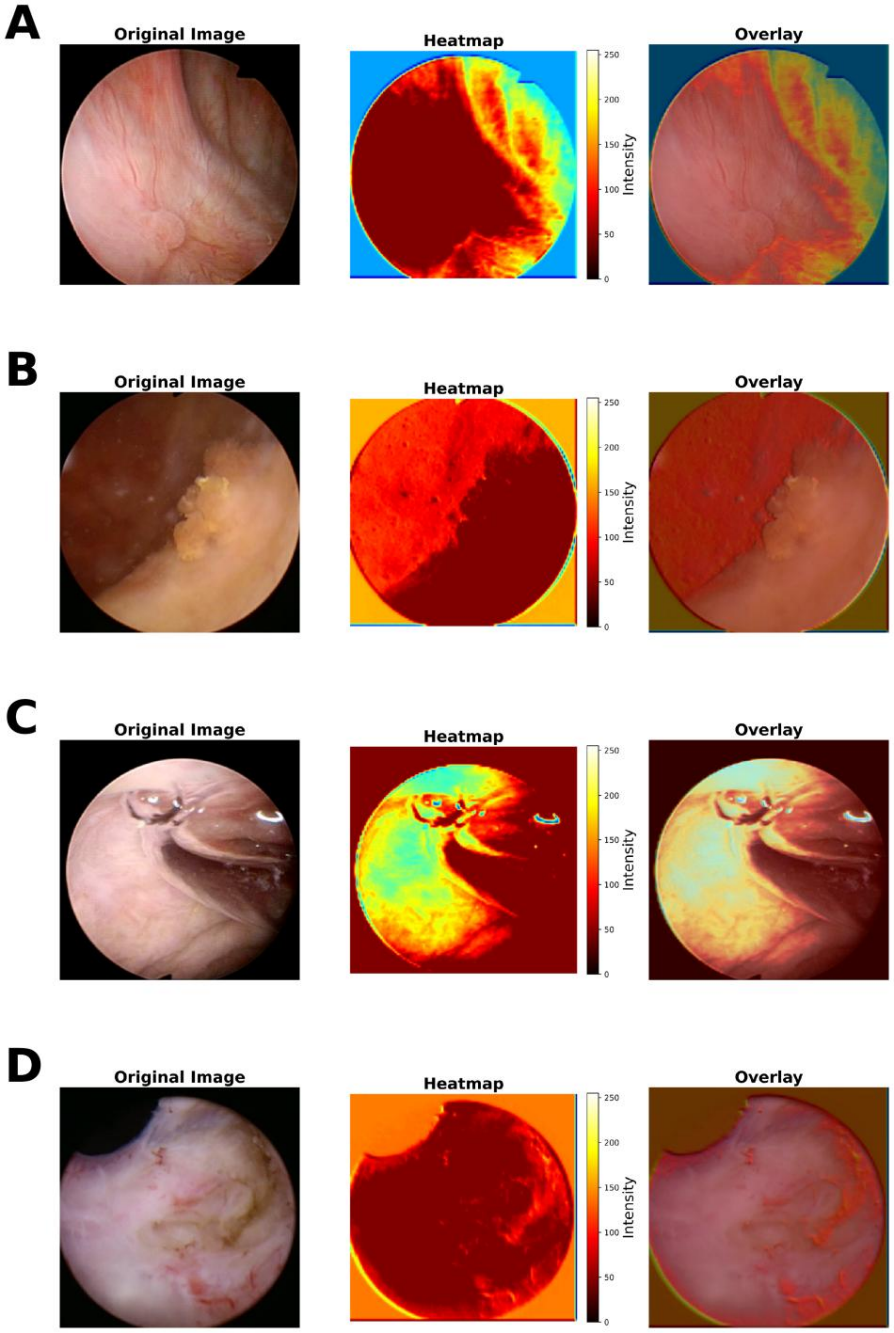
Visualization of image, heatmap, and overlay for all four class samples. (A) Visualization of image, heatmap, and overlay for a single HGC class sample. (B) Visualization of image, heatmap, and overlay for a single LGC class sample. (C) Visualization of image, heatmap, and overlay for a single NST class sample. (D) Visualization of image, heatmap, and overlay for a single NTL class sample.

Heatmaps were generated for model interpretability. It was done by leveraging Grad-CAM to visualize class-specific activations and assess model focus regions in the input images. It involved visualizing the areas of an image that contribute most to the model’s prediction by analyzing the activations and gradients of the model’s last convolutional layer. The figures are provided in Fig. 11. Four subfigures are illustrated, belonging to each of the four classes. The overlay image is also displayed, created by superimposing the original image and the heatmap.

## 4 Discussion and conclusion

This study presents DeepBCTPred, a novel dual-branch deep learning model for bladder cancer tissue prediction that achieves superior performance compared to existing state-of-the-art methods. Our model demonstrates exceptional classification accuracy with 98.74% recall, 99.45% specificity, 97.96% F1-score, 97.38% MCC, and 99.61% AUPR on an independent test dataset containing images across four classes (HGC, LGC, NST, and NTL). For the finalized model, DeepBCTPred was trained on the D4 set that had 550 HGC images, 790 LGC images, 591 NST images, and 147 NTL images. There was no overlap between the training and testing datasets in any of the evaluations, ensuring a fair assessment of model performance and preventing data leakage.

The model’s innovative architecture combines a MobileNetV3-based CNN for spatial feature extraction with a feedforward neural network processing handcrafted features, creating a synergistic approach that captures both local patterns and high-level statistical properties. Our comprehensive ablation studies confirm that this dual-branch design significantly outperforms individual components, demonstrating the clear value of integrating learned and engineered features. The genetic algorithm-based data augmentation strategy effectively addresses the challenge of limited medical imaging data, while recursive feature elimination ensures optimal model efficiency.

The clinical significance of this work lies in its potential to enhance diagnostic accuracy and reduce misdiagnosis in bladder cancer detection. The model generates interpretable heatmaps that highlight decision-critical regions, enabling pathologists to validate AI predictions and make more informed diagnoses. The consistent performance across all tissue classes, despite dataset imbalance, indicates robust generalization capability suitable for real-world clinical deployment. This is particularly important given that the independent dataset used has already been clinically validated for bladder cancer prediction, ensuring its clinical relevance and reliability.

While our model demonstrates promising diagnostic performance, several directions remain open for future investigation. One avenue involves incorporating pixel-level segmentation methods—such as U-Net or attention-based models—to localize cancer-associated regions more precisely, which may reduce background noise and improve feature relevance. This would, however, require expert-annotated region masks. Another potential direction is conducting prospective multi-center clinical trials to assess the model’s generalizability and clinical applicability. Developing clinician-friendly interfaces and establishing integration protocols could support real-world deployment within existing pathology workflows. Additionally, the architecture may be extended to other urological cancers or adapted for pan-cancer classification tasks.

Despite its strong performance, this study has some limitations. First, although DeepBCTPred was trained and evaluated on a clinically validated dataset, the dataset size remains relatively small compared to large-scale benchmarks in computer vision. Finally, while interpretable heatmaps were generated, the explainability of deep learning models remains an ongoing challenge, and further validation with pathologists is needed to ensure trust and usability in practice.

DeepBCTPred represents a significant advancement in AI-assisted bladder cancer diagnosis, demonstrating that the strategic combination of handcrafted and learned features can achieve state-of-the-art performance. The model’s robust architecture, comprehensive evaluation, and clinical applicability position it as a valuable tool for improving diagnostic accuracy and patient outcomes in bladder cancer management. The training, validation, and test scripts are freely available at https://github.com/nafcoder/DeepBCTPred, supporting broader adoption and continued research advancement in this critical healthcare domain. It is our sincere hope that our proposed model will significantly contribute to the research fields related to bladder cancer tissue prediction and ultimately improve patient care.

## Supplementary Material

vbag087_Supplementary_Data

## Data Availability

Datasets and Python scripts to reproduce the results are provided at https://github.com/nafcoder/DeepBCTPred. The training, validation, and test scripts are also made available.
